# Use of retrograde intrarenal surgery (RIRS) compared with mini-percutaneous nephrolithotomy (mini-PCNL) in pediatric kidney stones

**DOI:** 10.1007/s00345-022-04186-x

**Published:** 2022-10-16

**Authors:** Mahmoud Ahmed Mahmoud, Amir Samuel Shawki, Hany Mostafa Abdallah, Diaa Mostafa, Hossam Elawady, Mohamed Samir

**Affiliations:** Ain Shams University Hospital, Cairo, Egypt

**Keywords:** Cost-effectiveness, Pediatric stones, Mini-percutaneous nephrolithotomy, Retrograde intrarenal surgery, Complications

## Abstract

**Objective:**

We aimed to compare the cost-effectiveness and safety of retrograde intrarenal surgery (RIRS) and mini-percutaneous nephrolithotomy (mini-PCNL) for pediatric kidney stones management.

**Patients and methods:**

Ninety pediatric patients with single or multiple renal stones 1–3 cm in diameter were collected prospectively and equally divided into two groups to undergo RIRS or mini-PCNL. The groups were compared for fluoroscopy and operative time, postoperative hospital stay time, stone-free rate (SFR), need for auxiliary procedures, and cost as well as complications.

**Results:**

There were no differences found between RIRS and mini-PCNL groups with regard to operative time and postoperative DJ stent application, while the mean of fluoroscopy time and postoperative hospital stay was significantly shorter in the RIRS than in the mini-PCNL group. The SFR, auxiliary treatment on residual stones, and complications were comparable. In both groups, no major (Clavien IV–V) complications were observed. The mean cost of RIRS was $1210 and $733 for the mini-PCNL.

**Conclusions:**

Both RIRS and mini-PCNL are effective and safe treatment modalities for pediatric renal stones 10–30 mm in size. However, mini-PCNL is more cost-effective making it a viable alternative to RIRS.

## Introduction

The incidence of renal stones among children has increased significantly [1, 2]. Generally, congenital anatomical deformities, metabolic disorders, and recurrent urinary tract infections lead to renal stones in the childhood age population. This is why pediatrics are at higher risk of recurrent urolithiasis formation and may undergo multiple surgical interventions [3].

Extracorporeal shock wave lithotripsy (ESWL) has been traditionally used for the treatment of pediatric renal stones < 2 cm. It has major limitations like multiple sessions, requirement for anesthesia, steinstrasse formation, low stone‑free rates (SFR), and the possibility of injury to the growing kidney [4].

Recently, the use of both retrograde intrarenal surgery (RIRS) and percutaneous nephrolithotomy (PCNL) has increased in the surgery of renal stones because of their minimal invasiveness nature [5]. Mini-percutaneous nephrolithotomy (Mini-PCNL) is a modification of the standard PCNL maneuver that decreases the tract size and has gained widespread use as a pediatric endourological technique due to its fewer complications than the usual PCNL [6, 7].

However, even mini-PCNL may cause problems in the pediatric age group, because of the relatively smaller size and higher mobility of children's kidneys [8, 9]. Also, multiple accesses may be required for multiple stones management with the more added risk of complications [10]. A viable option for these patients is RIRS, which can be done at one or multiple stages according to stone number, size, and site [11]. While adult RIRS is considered now a well-established procedure to manage upper urinary tract stones with excellent outcomes, studies discussing its use in the pediatric age group are relatively sparse [12].

Nowadays, there is a need for selecting the best treatment option with the lowest cost and morbidity. There are a limited number of cost-effectiveness studies comparing the two modalities of therapy, especially in the pediatric age-group. In our study, we examined our experience comparing RIRS and mini-PCNL for children with 1- to 3-cm renal stones as regards cost-effectiveness and safety.

## Patients and methods

### Study population

This was a prospective clinical study, which was done between January 2019 and January 2022 in a single tertiary care center. Approval was obtained from the ethical committee of our institution before the start of the research (numbered FWA 000017585). After explaining the study steps including the possible side effects, written informed consent was signed by the parents.

Inclusion criteria were patients aged 3–14 years and had single or multiple renal stones 1–3 cm in diameter. Exclusion criteria were previous stone treatment, renal abnormalities, ureteral stones accompanied by renal stones, staghorn stones, and uncontrolled bleeding tendency.

Using the STATA program, adjusting alpha error at 5% and power at 80%. Results from a previous study by Lee et al. 2015 showed that 57% of the mini-PCNL cases had complications compared to 85% in RIRS cases. Based on this, the required sample size is 45 cases per group taking into consideration the 10% dropout rate [13].

**Fig. 1:**
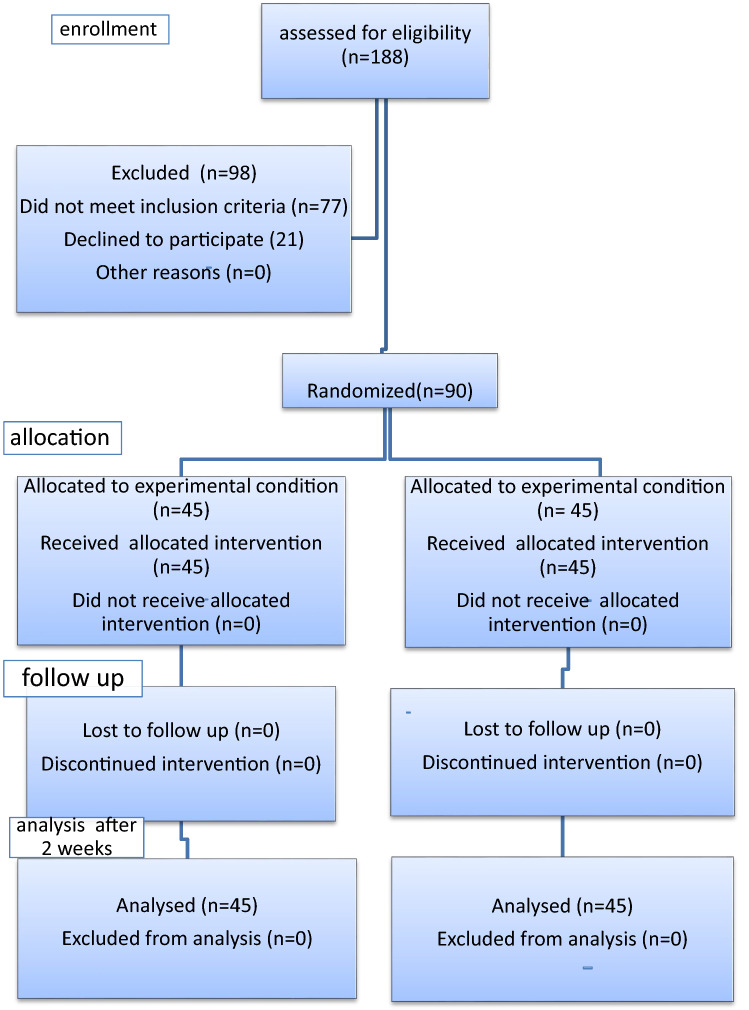
Consort flow chart

A total of 188 pediatric patients with renal stones were examined for eligibility to be involved in the study. Ninety-eight patients were excluded for these reasons: 77 patients were excluded for not meeting inclusion criteria: 68 with stone size outside our study inclusion range, 3 with previous stone treatment, 4 with ureteral stones accompanied by renal stones and 2 with renal abnormalities, while 21 declined to participate in the study, as shown in Fig. 1.

The remaining 90 patients using the sealed envelope method were randomly divided into 2 groups, each formed of 45 patients. Group A underwent RIRS and group B underwent mini-PCNL.

All patients were preoperatively assessed for complete blood count (CBC), coagulation profile, blood urea nitrogen (BUN), serum creatinine (Cr), phosphorus, calcium, parathyroid hormone, urine culture, and 24-h urine sample. Plain X-ray kidney ureter bladder (KUB), pelviabdominal-ultrasonography (USG), and non-contrast computed tomography (NCCT) were used as imaging methods in all patients. Stone size was measured as the longest diameter calculated on NCCT and in the presence of multiple stones as the sum of the longest diameters of all stones.

We investigated the efficacy of the approach by fluoroscopy and operative time, postoperative hospital stay time, SFR, need for auxiliary procedures, and cost as well as safety through fever, sepsis, bleeding, urinary leakage, and renal pelvis perforation.

SFR was defined as no or stone fragments < 4 mm in NCCT 1 month postoperatively. Postoperative complications were defined and graded using the Clavien–Dindo system [14]. The costing for the included patients was done at 2022 rates. Costs in Egyptian pounds were converted into USD. The cost of every procedure has computed as the sum of hospitalization, operating room use, fluoroscopy time, operative and anesthesia charges, the mean cost of the repair of the instrument, the cost of auxiliary procedures, and management of complications if needed. The primary endpoint was to evaluate the cost-effectiveness, and the secondary endpoint was to evaluate the safety of both procedures.

### Technique

All procedures were done under general anesthesia by the same expert surgical team, and stones were fragmented with a Holmium: YAG laser (272 mm holmium laser fiber).

- *RIRS* Patients were placed in the lithotomy position. After ureteral dilatation, a 9.5-Fr ureteral access sheath (UAS) was placed except in those with too thin urethras. Through the UAS, a 7.5-F flexible ureteroscope (FURS, Karl Storz, Tuttlingen, Germany). In case of failure of placement of the UAS, FURS was applied directly over the guide wire. Passive dilatation of the ureter for 2–4 weeks was done with double-J (DJ) if necessary. (FURS could not be advanced to the collecting system with or without UAS.) In all cases, fragmentation of the stones was done. Lower pole stones’ repositioning was attempted with a basket to be placed in the pelvis in some patients. We placed a postoperative DJ stent for 2–4 weeks if any visible ureteral or pelvicalyceal trauma occurred, significant fragmented stone burden at the end of the operation, or placement of the UAS for more than 45 min. Otherwise, a ureteral catheter was inserted and removed one day later.
- *Mini-PCNL* All procedures were done in the prone position. The target calyx is punctured using fluoroscopic guidance with an 18-GA sheathed needle, and tract dilatation was achieved using Amplatz dilators up to 16–18 Fr according to patient age. Standard 12-Fr rigid nephroscope was used for stone retrieval and fragmentation. A clamped nephrostomy tube was placed routinely to reduce the bleeding for 24 h in all cases. The nephrostomy tube and the ureteral catheter were removed on first and second postoperative days if there were no complications.

### Outcome measurements

Patient’s demographic data, stones parameters, fluoroscopy and operative time, complications, postoperative hospital stay time, SFR, auxiliary procedures if needed and mean costs were recorded and compared between the two groups.

### Statistical analysis

Data were fed to the computer and analyzed using IBM SPSS software package version 20.0. (Armonk, NY: IBM Corp). The Kolmogorov–Smirnov test was used to verify the normality of the distribution of variables, Paired *t*-test was used to compare two periods for normally distributed quantitative variables, while ANOVA with repeated measures was used for comparing the different studied periods for normally distributed quantitative variables and followed by post hoc test (Bonferroni-adjusted) for pairwise comparison. Pearson coefficient was to correlate between two normally distributed quantitative variables. The significance of the obtained results was judged at the 5% level.

## Results

There was no statistically significant difference found between RIRS and mini-PCNL groups regarding age, gender, body mass index (BMI), complaint at admission, stone size, and stone density, as shown in Table 1.

**Table 1:** Demographic data and complaints of the studied patients

	RIRS	Mini-PCNL	*P*-value
*No. of cases*	45	45	
*Age, year*	8.67 ± 2.73	9.22 ± 2.68	0.333
*Gender, n (%)*			
Male	21 (46.7%)	23 (51.1%)	0.673
Female	24 (53.3%)	22 (48.9%)	
*BMI, kg/m*^*2*^	19.91 ± 1.93	20.13 ± 2.19	0.611
*Complaint, n (%)*			
Hematuria	20 (44.4%)	28 (62.2%)	0.205
UTI	11 (24.4%)	9 (20.0%)	
Pain	14 (31.1%)	8 (17.8%)	
*Stone side*			
Right	15 (33.3%)	31 (68.9%)	
Left	30 (66.7%)	14 (31.1%)	
*Stone size, cm*	1.94 ± 0.70	2.04 ± 0.63	0.478
*Stone density, HU*	784.44 ± 248.59	803.38 ± 236.55	0.712

With regard to operative time and postoperative DJ stent application, there were no differences found between the RIRS and the mini-PCNL group. While the mean of fluoroscopy time and postoperative hospital stay was significantly shorter in the RIRS than in the mini-PCNL group. The UAS was placed in 15 (33.3%) patients of the RIRS group, as shown in Table 2. In the RIRS group, 18 (40%) children required preoperative DJ stent application to passively dilate the ureteric orifice.

**Table 2:** Comparison between RIRS group and mini-PCNL groups regarding perioperative data

	RIRS	Mini-PCNL	*P*-value
	No. = 45	No. = 45	
*Operative time, min*	88 ± 34.27	89.11 ± 33.58	0.877
*Fluoroscopy time, s*	93.16 ± 35.15	127.56 ± 54.97	0.001
*Hospital stay, h*	36.27 ± 11.0	55.60 ± 37.75	0.001
*Postoperative DJ application, n (%)*			
No	26 (57.8%)	33 (73.3%)	0.12
Yes	19 (42.2%)	12 (26.7%)	
*UAS placement, n (%)*			
No	30 (66.7%)		
Yes	15 (33.3%)		

Concerning initial SFR, auxiliary treatment on residual stones, and complications, there were no significant differences. The additional procedures increased the final SFR from 88.9 and 95.6% in RIRS and mini-PCNL groups, respectively, to 100%. In both groups, no major (Clavien IV–V) complications were observed, as shown in Table 3.

**Table 3:** Comparison between RIRS group and Mini PCNL group regarding outcome and postoperative data

	RIRS	Mini-PCNL	*P*-value
	No. = 45	No. = 45	
*Initial SFR, n (%)*			
Free	40 (88.9%)	43 (95.6%)	0.238
Residual	5 (11.1%)	2 (4.4%)	
*Auxiliary treatment on residual stones, n (%)*			
No	40 (88.9%)	43 (95.6%)	0.48
RIRS	2 (4.4%)	1 (2.2%)	
ESWL	2 (4.4%)	0 (0.0%)	
Chemo dissolution	1 (2.2%)	1 (2.2%)	
*Complication rate, n (%)*			
No	42 (93.3%)	38 (84.4%)	0.401
Postoperative fever (Grade II)	2 (4.4%)	2 (4.4%)	
Sepsis (Grade II)	0 (0.0%)	1 (2.2%)	
Postoperative hematuria (Grade I)	1 (0.0%)	2 (4.4%)	
Postoperative hematuria required blood transfusion (Grade II)	0 (0.0%)	1 (2.2%)	
Renal pelvis perforation (Grade III B)	0 (0.0%)	1 (2.2%)	

The mean cost of RIRS was $1210 and $733 for the mini-PCNL. Cost-effectiveness was measured using incremental cost-effectiveness ratio (ICER) according to the following equation: cost of RIRS – cost of Mini-PCNL/SFR of RIRS – SFR of Mini-PCNL = 1210–733/88.9–95.6 = – 71.1. This means that mini-PCNL was more cost-effective than RIRS, as shown in Table 4.

**Table 4:** Comparison between RIRS group and Mini PCNL group regarding cost

	RIRS	Mini PCNL	P-value
	No. = 45	No. = 45	
*Average cost per case*			
Mean ± SD	1210 ± 151.25	733 ± 91.63	< 0.001
Range	950–1320	610–1120	< 0.001
*Operating room fees*	379 ± 47.38	205 ± 25.63	< 0.001
*Anesthesia charges*	52 ± 6.50	51 ± 6.38	0.463
*Surgical equipment used*			
LASER FIBERs per 10 cases in average	**1049 ± 100**	**1037 ± 105**	0.580
Irrigation fluid 3.5 $ per L	12 ± 1.5	21 ± 2.63	< 0.001
Basket	92 ± 11.5	–	–
DJ	22 ± 2.75	22 ± 2.75	1.000
Nephrostomy tube	–	0.21 ± 0.03	–
Fluoroscopy	15 ± 1.88	23 ± 2.88	< 0.001
*Mean cost of the repair of the instrument*			
Cost of repair of flexible URS used every 10 cases	**615 ± 76.88**	–	–
No. of repairs of flexible URS needed	5 ± 1	–	–
Cost of repair of pediatric nephroscope	–	–	–
*Postoperative costs*			
Antibiotic	5 ± 0.63	6 ± 0.75	< 0.001
Radiology test	7.3 ± 0.91	7.7 ± 0.96	0.045
Laboratory test	3 ± 0.38	4 ± 0.50	< 0.001
*Auxiliary procedures*			
RIRS	395 ± 49.38	370 ± 46.25	0.015
ESWL	100 ± 12.5	–	–
Chemodissolution	17 ± 2.13	15 ± 1.88	< 0.001
*In-hospital complications*			
Blood transfusion	–	31 ± 3.88	–
*Hospital stay*	90 ± 11.25	150 ± 18.75	< 0.001

## Discussion

In 1985, the initial percutaneous stone removal in children was performed using adult instruments. In these series, the incidence of complications and blood transfusion was higher but with a similar success rate [15, 16]. In 1998, Jackman et al. popularized the first mini-PCNL series to reduce the higher complication rates. They performed 11 procedures using 11-Fr sheath and 7-Fr rigid cystoscope and reported no complications [17]**.**

The prevalence of urolithiasis below 18 years is about 1–2% [18]. Although pediatric urolithiasis is an uncommon health problem, these patients may need multiple surgical interventions due to recurrent stone disease. For this reason, minimal invasive modalities like ESWL, RIRS, mini-PCNL, and micro-PCNL have become important management options for pediatric stones [19]. However, there are no enough data in the literature regarding the comparison of these modalities in pediatric patients.

In our study, there were no statistically significant differences in either group regarding operative time and postoperative DJ stent application. However, fluoroscopy time and postoperative hospital stay were significantly shorter in the RIRS than in the mini-PCNL group (93.16 ± 35.15 s vs 127.56 ± 54.97 s) and (36.27 ± 11.0 h vs 55.60 ± 37.75 h), respectively. This was in agreement with the results of Pelit et al. who reported that fluoroscopy and hospitalization times were statistically higher in the mini-PCNL group than in the RIRS group. They attributed the longer hospital stay to the presence of a nephrostomy tube and more ongoing postoperative pain in the mini-PCNL group [19]**.** Also, Ferroud et al. found out that the postoperative hospital stay in the RIRS group was significantly shorter than in the mini-PCNL group with a mean hospital stay time of 1.49 ± 11.4 days compared to 4.1 ± 1.2 days, respectively (*P* < 0.05) [20]**.**

The application of UAS reduces the operative time, intrarenal pressure, and its associated complications and increases SFR, but its use is still debated widely [12]**.** In the present study, the UAS has placed in 15 (33.3%) patients of the RIRS group without any complications. This agreed with Karunakaran et al.’ work that found no UAS-related complications but with prestenting of all the children below 5 years [12]. On the other side, in a series of 96 children with a mean age of 13 years, authors stated that 7.3% of patients had UAS-associated complications [21]. Also, Schuster et al. noted that only 2 of 221 ureteroscopies in children suffered from strictures and 8 developed vesicoureteral reflux of low grade [22]. This can be explained by the larger study population in their work and prestenting of any child with the too thin urethra or usage of FURS directly in UAS application failure in our study.

The outcome of RIRS relies on the stone burden, and it decreases significantly in stone size above 20 mm. Many studies concluded that RIRS is more effective than mini-PCNL in stones smaller than 20 mm and mini-PCNL has a superior outcome with bigger stones, while RIRS still represents an alternative to it [23]. With regard to the initial SFR, we found no significant differences between the two groups (88.9% and 95.6% in the RIRS and mini-PCNL groups, respectively). Also, both procedures were comparable regarding the need for auxiliary treatment on residual stones. This matches with Resorlu et al.’s comparative analysis of RIRS and mini-PCNL in the management of pediatric renal stones 10–30 mm in size. They noted SFR of 84% and 86% for RIRS and mini-PCNL groups, respectively. They concluded that the RIRS success rate decreases markedly with stone size above 20 mm [24]. Also, Zhang et al. reported that the SFR achieved by RIRS for stones larger than 2 cm was 79.7% and 80.9% after mini-PCNL [25]. However, Pelit et al. found out that for pediatric stones the SFR of RIRS (75%) was less than the SFR of mini-PCNL (84.4%) [19].

With regard to the complications, our study reported no significant difference between the two groups. We documented mainly grade I and II complications in both groups except for 1 patient in the mini-PCNL group that suffered from renal pelvis perforation (grade III B) and was managed successfully with stent application. No major (grade IV–V) complications were observed in either group. This is in agreement with Resorlu et al. who reported that the total complications in mini-PCNL were more but this difference was not statistically significant [24]. Also, Pelit et al. observed only minor complications (grade I, II, and III) happened in 8.4% and 17% of RIRS and mini-PCNL, respectively, but again the difference was not statistically significant [19].

There is a persistent demand for providing value-based care, especially with the ongoing economic pressures, so there is a must to assess the cost-effectiveness of new procedures prior to their usage [26]. In the present study, the mean cost per procedure was higher in the RIRS group than in the mini-PCNL group. This can be explained by the high cost of repair of FURS. Going with this, Bagcioglu et al. and Erdoğan et al. reported that the cost per patient was significantly lower in PCNL than in RIRS group. They explained the need for auxiliary equipment and expensive materials in RIRS and their short lifetime [27, 28]. On the contrary, Wymer et al. found that RIRS was a more cost-effective procedure than ESWL and PCNL for renal stones 1–2 cm in size. They attributed that to the relatively high SFR and low complication rate of RIRS reported in their research [29].

The main limitations of our study were: we did not assess stone chemical composition as we used laser for stone disintegration, postoperative pain scores, and stone volume. However, we think that our findings will contribute well to the continuous research regarding this critical topic.

## Conclusion

Both RIRS and mini-PCNL are effective and safe treatment modalities for pediatric renal stones 10–30 mm in size. However, mini-PCNL is more cost-effective making it a viable alternative to RIRS.
